# Bufexamac ameliorates LPS-induced acute lung injury in mice by targeting LTA4H

**DOI:** 10.1038/srep25298

**Published:** 2016-04-29

**Authors:** Qiang Xiao, Ningning Dong, Xue Yao, Dang Wu, Yanli Lu, Fei Mao, Jin Zhu, Jian Li, Jin Huang, Aifang Chen, Lu Huang, Xuehai Wang, Guangxiao Yang, Guangyuan He, Yong Xu, Weiqiang Lu

**Affiliations:** 1 The Genetic Engineering International Cooperation Base of Chinese Ministry of Science and Technology, Key Laboratory of Molecular Biophysics of Chinese Ministry of Education, College of Life Science and Technology, Huazhong University of Science & Technology, Wuhan 430074, China; 2 Shanghai Key Laboratory of New Drug Design, School of Pharmacy, East China University of Science and Technology, Shanghai 200237, China; 3 Hubei Bio-pharmaceutical Industrial Technological Institute Inc., Wuhan 430075, China; 4 Shanghai Key Laboratory of Regulatory Biology, Institute of Biomedical Sciences, School of Life Sciences, East China Normal University, Shanghai 200241, China

## Abstract

Neutrophils play an important role in the occurrence and development of acute lung injury (ALI). Leukotriene B4 (LTB4), a hydrolysis product of epoxide leukotriene A4 (LTA4) catalyzed by LTA4 hydrolase (LTA4H), is one of the most potent chemoattractants for neutrophil. Bufexamac is a drug widely used as an anti-inflammatory agent on the skin, however, the mechanism of action is still not fully understood. In this study, we found bufexamac was capable of specifically inhibiting LTA4H enzymatic activity and revealed the mode of interaction of bufexamac and LTA4H using X-ray crystallography. Moreover, bufexamac significantly prevented the production of LTB4 in neutrophil and inhibited the fMLP-induced neutrophil migration through inhibition of LTA4H. Finally, bufexamac significantly attenuated lung inflammation as reflected by reduced LTB4 levels and weakened neutrophil infiltration in bronchoalveolar lavage fluid from a lipopolysaccharide-induced ALI mouse model. In summary, our study indicates that bufexamac acts as an inhibitor of LTB4 biosynthesis and may have potential clinical applications for the treatment of ALI.

Acute lung injury (ALI) is a clinical syndrome of acute respiratory failure that frequently occurs in critically ill patients, with a high morbidity, and an overall mortality estimated at 46%, this syndrome has an extensive effect on public health^1^,^2^,^3^. ALI is clinically characterized by increased permeability of the alveolar-capillary barrier, thus leading to lung edema with pulmonary infiltrates and hypoxemia^4^,^5^. The pathophysiology of ALI involves a complicated imbalances between pro-inflammatory and anti-inflammatory cytokines, oxidants and antioxidants, neutrophil recruitment and activation and mechanisms of neutrophil clearance^6^. There are a number of risk factors associated with ALI, such as sepsis, pneumonia or multiple traumatic injuries, either in a direct manner or remote to the lung. To date, no Food and Drug Administration-approved drugs can be used to control ALI. Thus, identifying novel therapeutic agents and strategies is urgent.

Neutrophils are the earliest immune cells to be recruited into the site of injury or inflammation, followed by the increase of alveolar-capillary barrier permeability and lung edema. Thus, the activation and transmigration of neutrophils are involved in the progression of ALI. Activated neutrophils are able to extravasate and migrate into the alveolar space, mediated by a complex interaction of chemokines^5^. After migration, neutrophils can release granule proteins (proteolytic enzymes and cationic peptides) and reactive oxygen species (ROS), which may cause further damage to the endothelium and epithelium. Leukotriene B4 (LTB4) can be produced by neutrophils and has potent chemotactic activity, which indicate that LTB4 may be a significant mediator for the migration of neutrophils in ALI^7^,^8^. LTB4 is a metabolite of arachidonic acid (AA) in which leukotriene A4 (LTA4) hydrolase (LTA4H) catalyzes the final step in LTB4 biosynthesis.

Bufexamac, an arylalkanoic acid derivative, is a non-steroidal anti-inflammatory drug (NSAID) for topical treatment of eczema and other inflammatory dermatoses^9^. The bufexamac cream was reported to have an anti-inflammatory effect comparable to fluocinolone acetonide cream^10^,^11^. However, the exact mechanism of the anti-inflammatory role of bufexamac is still unclear. In the present study, we first report that bufexamac is a selective inhibitor of LTA4H, a crucial enzyme in the LTB4 biosynthesis pathway. Furthermore, bufexamac is able to reduce LTB4 biosynthesis, inhibit neutrophil chemotaxis, and ameliorate the lipopolysaccharide (LPS)-induced ALI in mice through targeting LTA4H. The protective effect of bufexamac on LPS-induced ALI via LTB4 biosynthesis may provide a new therapeutic approach for ALI patients.

## Results

### Bufexamac selectively targets LTA4H

**Figure 1:**
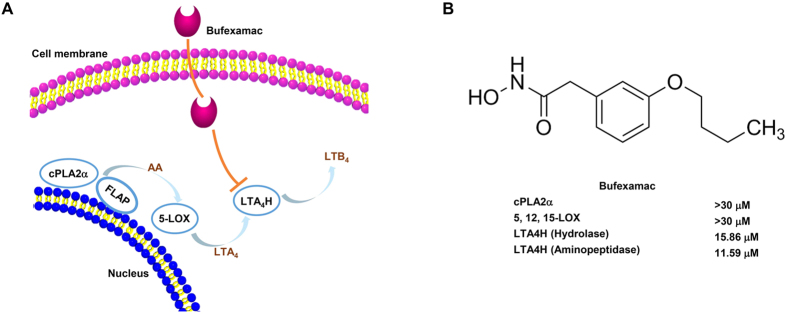
Bufexamac is selective inhibitor of LTA4H. The *in vitro* inhibitory activities of bufexamac against cPLA2α, LOXs and LTA4H were determined as described in the method. Bufexamac exhibits specific inhibition on LTA4H epoxide hydrolase and aminopeptidase activities.

The inhibition activity of bufexamac against enzymes in the lipoxygenase (LOX) pathway was determined to investigate the effect of bufexamac on the LTB4 biosynthesis pathway systematically. As shown in Fig. 1A, the production of LTB4 was catalyzed by a series of enzymes, such as cPLA2α, 5-LOX and LTA4H. Bufexamac significantly inhibited the activities of LTA4H aminopeptidase and epoxide hydrolase with the IC_50_ values of 15.86 and 11.59 μM, respectively (Fig. 1B). However, bufexamac had only a slight effect on the enzymatic activities of cPLA2α and 5-LOX, which also play key roles in LTB4 biosynthesis. In addition, two important arachidonic acid metabolic enzymes, 12-LOX and 15-LOX, were unperturbed by bufexamac. These data indicate that bufexamac specifically inhibited LTA4H activity, which may subsequently influence the production of LTB4.

### Bufexamac binds to the catalytic zinc of LTA4H

**Figure 2:**
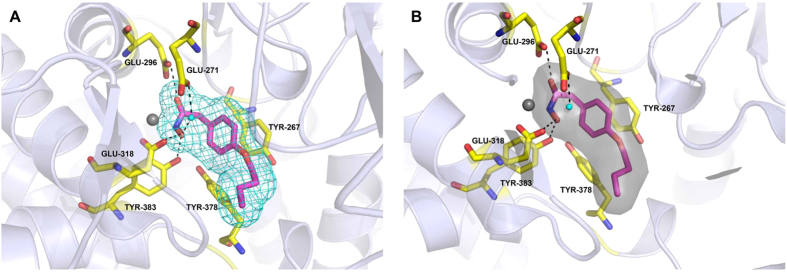
Crystal structure of LTA4H in complex with the bufexamac. X-ray structure of human LTA4H bound to bufexamac solved to 2.03 Å resolutions. Ribbon representation of LTA4H is shown in lightblue. (**A**) 2Fo−Fc electron density map contoured at 2.0σ showing the compound binding site on LTA4H (cyan mesh and gray surface contoured at 2.0σ and 3 Å around the ligand). (**B**) Bufexamac and neighboring protein environment are shown as thin stick model. Zinc ions are represented as gray spheres. Black dashed lines presented the hydrogen bonds. The water molecule is depicted as a cyan ball. The carbonyl and hydroxyl group of bufexamac bind to three key residues (Glu296, Glu318 and Tyr383, respectively) and the active site Zn^2+^ of LTA4H by hydrogen bonds. Besides, bufexamac interacts with Tyr267 by a pi-pi interaction.

A high resolution crystal structure of LTA4H complexed with bufexamac was resolved to reveal the interaction between bufexamac and LTA4H. As shown in Fig. 2, during the hydrolysis of LTA4 into LTB4, the Zn^2+^ of LTA4H binds to the 5, 6-epoxide moiety of LTA4 and initiates the chemical reaction in which the introduction of water occurs distant (C12) from the epoxide ring (C5/C6) and a unstable *cis* double bond is generated at ∆6 (between C6 and C7) in LTB4^12^,^13^. The aminopeptidase activity of LTA4H follows a general base mechanism. Structural elucidation of the interaction of bufexamac with LTA4H revealed that the carbonyl and hydroxyl groups of bufexamac were bound to three key residues (Glu296, Glu318 and Tyr383) and the Zn^2+^ in the active site of LTA4H, blocking the access to the substrate channel of the active site. Bufexamac also interacted with Tyr267 by a pi-pi interaction.

### Bufexamac significantly reduces LTB4 biosynthesis in neutrophil

**Figure 3:**
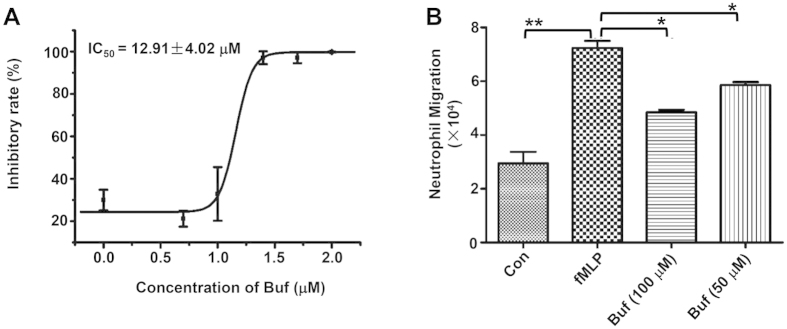
Bufexamac inhibits LTB4 biosynthesis in neutrophil and reduces neutrophil migration. (**A**) Isolated neutrophils were pretreated with indicated concentrations of bufexamac for 30 min, then stimulated with 5 μM A23187 for 30 min. The LTB4 concentration was measured by a commercial LTB4 EIA kit. (**B**) Bufexamac reduced fMLP (5 μM) induced neutrophil migration in a transwell assay. Data are expressed as mean ± SD (n = 3). *P < 0.05, **P < 0.01, ***P < 0.001.

To further investigate the mechanism by which bufexamac affects neutrophilic inflammation, the release of LTB4 in neutrophil was measured. Neutrophil was isolated from mouse bone marrow and stimulated with the calcium ionophore A23187 to activate the arachidonic acid pathway and release LTB4. As shown in Fig. 3A, the release of LTB4 in neutrophil was inhibited by bufexamac in a dose-dependent manner (IC_50_ = 12.91 ± 4.02 μM).

### Bufexamac significantly inhibits neutrophil chemotaxis

LTB4 acts as a secondary chemoattractant in response to formyl peptides, and is important in amplifying the neutrophil recruitment and inflammation process^14^. We examined the effects of bufexamac on the directional migration of neutrophil using a transwell assay to see if bufexamac mediated LTB4 reduction plays a role in the migration of neutrophil. Neutrophil was isolated from bone marrow and purified for the transwell assay. Under the influences of a primary chemoattractant of neutrophil (fMLP), the number of neutrophil migrating into the lower chamber was increased by more than two fold. Meanwhile, the pretreatment of neutrophil with bufexamac significantly inhibited fMLP induced neutrophil migration (Fig. 3B). These results indicated that bufexamac might inhibit neutrophil chemotaxis *via* the inhibition of LTA4H-mediated endogenous LTB4 biosynthesis.

### Bufexamac ameliorates LPS-mediated lung histopathologic changes

**Figure 4:**
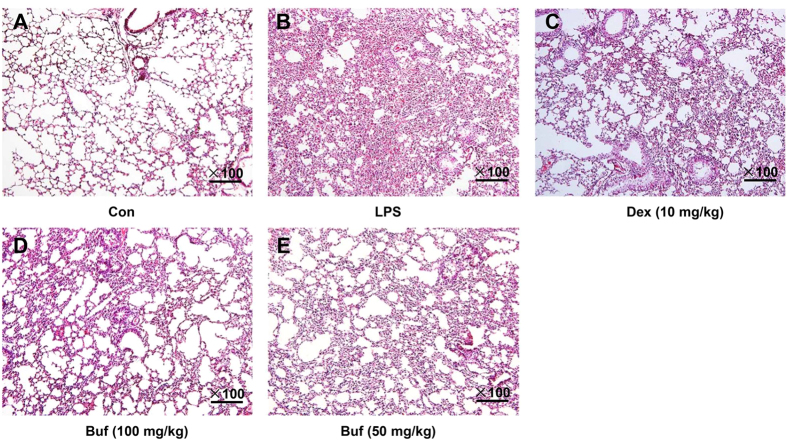
Bufexamac ameliorates LPS-mediated lung histopathologic changes. Lung tissue sections were stained with hematoxylin and eosin (H&E) for histopathology analysis. (**A**) Control, (**B**) LPS treated, (**C**) LPS and dexamethasone (10 mg/kg) treated, (**D**) LPS and bufexamac (100 mg/kg) treated; (**E**) LPS and bufexamac (50 mg/kg) treated.

The migration of neutrophil is usually a hallmark event in the progression of ALI. In the present study, LPS-induced ALI was utilized to evaluate the therapeutic effect of bufexamac *in vivo*. Lungs from each group of mice were collected at 24 h after the last LPS injection and stained with hematoxylin and eosin (H&E) to investigate the therapeutic effect of bufexamac on ALI. As shown in Fig. 4, influx of inflammatory cells into the alveolar space and thickening of the interalveolar septum were observed in LPS-induced mice compared with phosphatic buffer solution (PBS) treated mice. By contrast, bufexamac treatment significantly ameliorated the infiltration of lung inflammatory cells and injury to the lung in a dose-dependent manner.

### Bufexamac alleviates LPS-induced lung injury

**Figure 5:**
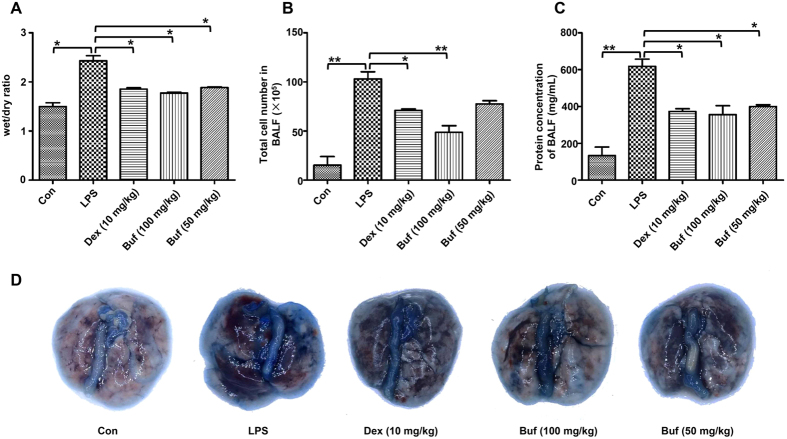
Bufexamac relieves LPS-induced lung injury. (**A**) Dissected lung tissues were weighed and oven-dried at 60 °C for 48 h for calculation of wet/dry ratio. (**B**) Total cells in BALF were counted using an automated cell counter (Invitrogen, Waltham, MA, USA). (**C**) The total protein concentration in supernatant was measured by BCA method. (**D**) Lung permeability was assessed by injecting Evans blue dye (50 mg/kg) into the caudal vein 1 h before euthanasia. Data are expressed as mean ± SD (n = 10 mice per group). *P < 0.05, **P < 0.01, ***P < 0.001.

Various parameters related to acute phase response, such as wet/dry weight ratio, total cells and proteins, and lung permeability, were assayed to evaluate the LPS-induced ALI. First, we measured the lung wet/dry weight ratio to evaluate the lung water content and edema. As presented in Fig. 5A, bufexamac partly reduced LPS-induced lung edema. Then, we measured the total cells and proteins in bronchoalveolar lavage fluid (BALF) (Fig. 5B,C), as a measure of the severity of lung permeability^15^. As shown in Fig. 5, bufexamac treatment significantly reduced LPS administration increased BALF cell numbers and protein levels.

Evans blue dye was injected into the caudal vein to evaluate the effect of bufexamac on lung permeability directly. One hour after the injection of Evans blue dye, the mouse was killed and its entire lung was extracted and photographed. As shown in Fig. 5D, LPS challenge increased lung vascular leakage. Meanwhile, less dye was extravasated in bufexamac-treated mice, indicating a protective effect of bufexamac on the alveolar-vascular barrier.

### Bufexamac inhibits neutrophil infiltration and reduces LTB4 level *in vivo*

**Figure 6:**
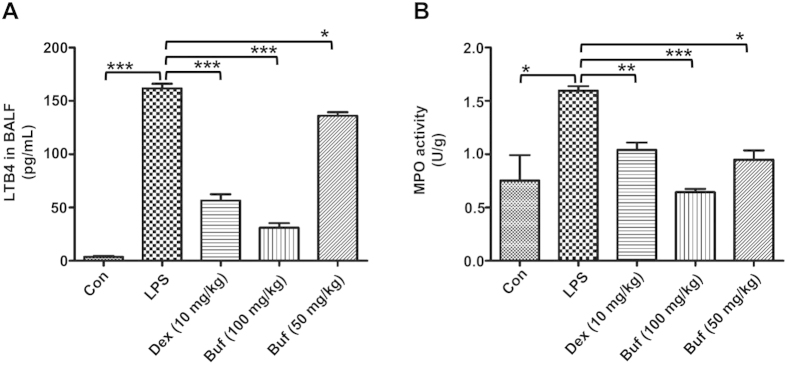
Bufexamac reduces LTB4 level and MPO activity. (**A**) LTB4 level in supernatant was quantified using a LTB4 EIA kit (Cayman, Ann Arbor, MI, USA). (**B**) MPO activity was measured in lung homogenates. Data are expressed as mean ± SD (n = 10 mice per group). *P < 0.05, **P < 0.01, ***P < 0.001.

The levels of LTB4 in the BALF of mice with LPS-induced ALI were measured by a commercial LTB4 EIA kit. As shown in Fig. 6A, LPS significantly increased the level of LTB4 in BALF. However, the elevated LTB4 levels were significantly reduced by bufexamac administration, which was consistent with the inhibition of LTB4 released by neutrophils *in vitro*. Neutrophil infiltration was then determined by measuring the myeloperoxidase (MPO) activity, which is often used as a marker of neutrophil infiltration. As shown in Fig. 6B, MPO activity in the lung homogenates of LPS-induced mice was approximately two fold that of the control group. Bufexamac significantly reduced the MPO activity in a dose-dependent manner. These results indicate that bufexamac has a favorable effect on preventing the migration of neutrophil into the alveolar space, which may be mediated by the decrease of LTB4 biosynthesis.

### Bufexamac reduces LPS-induced lung inflammatory cytokines

**Figure 7:**
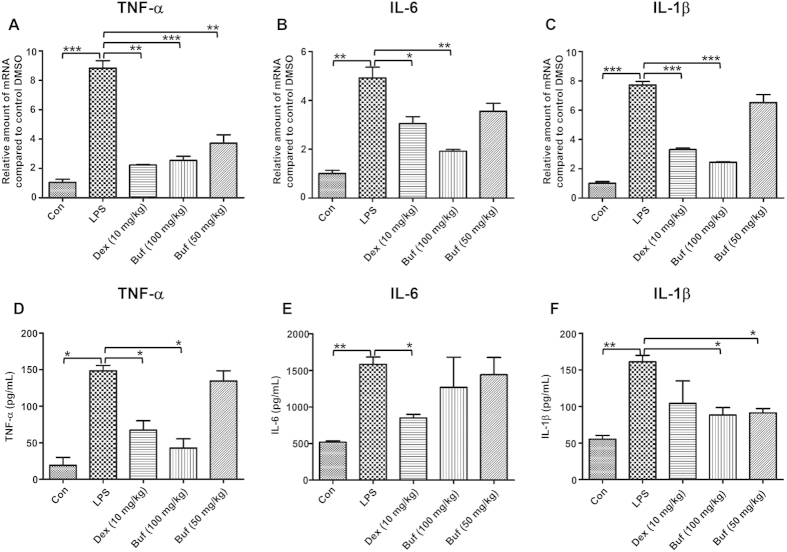
Bufexamac reduces cytokine mRNA expressions in lung tissue and cytokine levels in BALF in LPS-induced ALI in mice. Bufexamac reduced the mRNA expressions of pro-inflammatory cytokines, such as TNF-α (**A**), IL-6 (**B**), and IL-1β (**C**). Bufexamac reduced pro-inflammatory cytokines levels, including TNF-α (**D**), IL-6 (**E**) and IL-1β (**F**). Data are expressed as mean ± SD (n = 10 mice per group). *P < 0.05, **P < 0.01, ***P < 0.001.

Various inflammatory cytokines were known to be involved in LPS-induced ALI. To further investigate the protective effect of bufexamac in the LPS-induced lung inflammation, we measured the gene expression of proinflammatory cytokines, such as TNF-α, IL-1β and IL-6, in lung homogenates. As shown in Fig. 7, LPS administration significantly up-regulated the mRNA expression of TNF-α, IL-1β and IL-6, whereas bufexamac treatment effectively suppressed their up-regulation, particularly TNF-α. In addition, protein levels of these cytokines were relatively consistent with the mRNA levels. Bufexamac treatment significantly reduced the expression levels of TNF-α, IL-1β and IL-6.

## Discussion

Bufexamac is a NSAID for the topical treatment of eczema and other inflammatory dermatoses^9^. Bufexamac is also reported to possess anti-inflammatory properties in other inflammatory diseases, such as arthritis and eczema. It was reported that bufexamac ameliorated edema in carrageenan-induced arthritis in rats and relieved erythema in ultraviolet irradiation-induced skin rash in guinea pigs^16^,^17^. However, the exact mechanism of the anti-inflammatory effect of bufexamac is still unknown. In the present study, we demonstrated that bufexamac suppressed neutrophil chemotaxis by directly blocking the enzymatic reaction of LTA4H, a critical enzyme in LTB4 biosynthesis. The intermolecular interactions between LTA4H and bufexamac were revealed by a high-resolution complex crystal structure. Further investigation of bufexamac’s effect on neutrophilic inflammation disease indicated that bufexamac significantly reduced LTB4 level in LPS-induced ALI and ameliorated LPS-induced lung injury and inflammation.

We found that bufexamac selectively inhibited the catalytic activity of LTA4H, but had less inhibitory effect on other enzymes in the LTB4 biosynthesis pathway, such as 5-LOX, 12-LOX, 15-LOX and cPLA2α. LTA4H is a widely expressed bifunctional zinc metalloenzyme with epoxide hydrolase and aminopeptidase activities using the same Zn^2+^ in the active site. LTA4H generates the 12R epimer of the hydroxyl group and forms the Δ^6^-*cis*-Δ^8^-*trans*-Δ^10^-*trans* configuration of the conjugated triene during hydrolysis of LTA4 to LTB4^12^. The investigation into the structure of LTA4H complexed with bufexamac revealed that bufexamac competitively bonded to Zn^2+^ in the active site, thus preventing the approach of endogenous substrate LTA4.

Further investigation revealed that bufexamac markedly inhibited LTB4 biosynthesis in isolated neutrophil and fMLP induced neutrophils chemotaxis in a dose-dependent manner. These *ex vivo* results are confirmed in a mouse model of LPS-induced ALI with reduced LTB4 levels and reduced neutrophil activity following bufexamac treatment. In addition, bufexamac significantly reduced the production of pro-inflammatory cytokines (TNF-α, IL-1β and IL-6) in the LPS-induced ALI model. TNF-α and IL-1β are early response cytokines to LPS-induced acute injury and have been identified in BALF from patients with ALI and acute respiratory distress syndrome^5^. TNF-α and IL-1β could stimulate macrophages, endothelial cells, and epithelial cells to release other pro-inflammatory cytokines, such as IL-6, thus amplifying the inflammation process.

Thus far, LTB4 is known as one of the most potent chemokines for neutrophil^12^. LTB4 is a secondary chemoattractant secreted by neutrophil, and is important in initiating the inflammation process. Neutrophils are the first group of immune cells recruited into the inflammation site, and are the most abundant leukocytes in the blood stream. In response to primary chemoattractants such as fMLP, neutrophil secrete secondary chemoattractants, such as LTB4, and recruit more leukocytes to amplify the inflammation process. The influx of neutrophil into the interstitium and bronchoalveolar space is believed to play a key role in the progression of ALI. LTB4 was associated with pulmonary disorders and neutrophil migration in LPS-induced ALI. Blocking of the LTB4 receptor significantly ameliorated LPS-induced hypoxemia, pulmonary edema and alveolitis^8^, suggesting the suppression of the LTB4 activity may have potential clinical applications in the treatment of neutrophilic inflammation associated diseases, such as ALI.

Bufexamac was also reported to exhibit pro-inflammatory activity, which led to the withdraw of marketing licenses for preparations containing bufexamac as a treatment of pruritic dermatitis in 2010^18^. In this study, we observed that bufexamac exhibits dual inhibition activities against LTA4H aminopeptidase and epoxide hydrolase. LTA4H is a bifunctional zinc metallopeptidases, which cleaves the endogenous chemotactic Pro–Gly–Pro that has a role in aminopeptidase activity, leading to the resolution of inflammation, whereas the epoxide hydrolase activity of LTA4H plays a role in the generation of LTB4, a potent chemokine for neutrophils^19^. Thus, the unexpected inhibition of the aminopeptidase activity of LTA4H provides a novel insight into the mechanism of allergic contact dermatitis caused by bufexamac. Indiscriminately targeting LTA4H dual enzyme activities may account for the observed paradoxical effect of bufexamac on the treatment of contact dermatitis, although the potential effect of bufexamac on the LTB4 level and Pro–Gly–Pro degradation in patients warrant further clinical investigation.

There are two potential limitations in current study. First, we revealed protective effects of bufexamac on ALI in mice. However, the *in vivo* toxicity and pharmacokinetics of bufexamac are poorly defined as it is usually embedded in ointments and lotions. Further detailed pharmacokinetics study of bufexamac in anti-inflammation clinical trials may be warranted. Second, although the reduction of pro-inflammatory factors, including TNF-α, IL-6, and IL-1β was observed in this study, we did not decipher the molecular mechanism of the effect of bufexamac on these pro-inflammatory factors. Such an investigation was outside the scope of the current study. Recently, bufexamac was identified as a selectively HDAC6 inhibitor and caused tubulin hyperacetylation. Hence inhibition of HDAC6 may be also involved in reducing proinflammatory factors production in LPS-induced ALI model by bufexamac^20^. However, further characterization of LTB4’s involvement in bufexamac-induced pro-inflammatory factor down-regulation in the mouse model of ALI would be meaningful.

## Methods

### Protein expression and purification

Human 5-LOX, 12-LOX and 15-LOX were expressed and purified as described previously^21^. Human cPLA2α was cloned into pET28a and overexpressed in BL21(DE3)pLysS. Recombinant cPLA2α was purified by Ni^2+^ affinity chromatography for further enzymatic assay^22^,^23^. Human LTA4H was cloned into pET28a and overexpressed in BL21(DE3)pLysS. Recombinant LTA4H was purified by Ni^2+^ affinity chromatography as described previously^24^,^25^.

### LOX activity assay

The 5-LOX, 12-LOX and 15-LOX activities were determined as described previously^26^. In brief, the assay is based on the oxidation capacity of the products of LOX and the production of fluorescent probe rhodamine 123 was monitored by fluorescence spectroscopy at *Ex/Em* = 500/536 nm on the PerkinElmer EnVision Multilabel Plate Reader.

### cPLA2α activity assay

The enzymatic activity of cPLA2α was determined by monitoring the hydrolysis of 7-hydroxycoumarinyl-γ-linolenate continuously with excitation at 360 nm and emission at 460 nm^27^. In brief, 7-HCEase activity was measured in a sonicated 200 μL of 1,2-O-tetradecyl-sn-glycero-3-phosphocholine (DTPC)/Triton X-100 high-affinity mixed micelle solution containing 960 μM Triton X-100, 60 μM DTPC, 50 μM 7-hydroxycoumarinyl-γ-linolenate, 50 mM N-2-hydroxyethylpiperazine-N-2-ethane sulfonic acid buffer (pH = 7.2), 0.3 mM EDTA, 1 mM CaCl_2_ and 300 mM KCl. The formation of 7-hydroxycoumarin was monitored using the PerkinElmer EnVision Multilabel Plate Reader.

### LTA4H aminopeptidase assay

The enzymatic activity of LTA4H aminopeptidase was determined by monitoring the hydrolysis of Ala-*p*-NA as previously reported with a minor modification^28^. The enzyme (500 ng) was incubated with the indicated concentrations of bufexamac at room temperature for 10 min in 96-well plates in 50 mM Tris-HCl, pH 7.5, containing 100 mM NaCl. Ala-*p*-NA was added to initiate the reaction. Then, the formation of *p*-NA was monitored for 10 min at 405 nm using the PerkinElmer EnVision Multilabel Plate Reader.

### LTA4H epoxide hydrolase assay

The enzymatic activity of LTA4H epoxide hydrolase was determined by monitoring the hydrolysis of LTA4. The enzyme (30 ng) was incubated with the indicated concentrations of bufexamac at room temperature for 10 min in 96-well plates in 10 mM NaH_2_PO_4_/Na_2_HPO_4_, pH = 7.4, containing 2 mg/mL bovine serum albumin (BSA). LTA4 was added to initiate the reaction. The production of LTB4 was determined by a commercial LTB4 EIA kit (Cayman Chemical, Ann Arbor, MI, USA) according to the manufacturer’s instruction.

### X-ray crystallography

Bufexamac was co-crystallized with LTA4H by liquid-liquid diffusion. LTA4H (10–15 mg/mL) was incubated with bufexamac (2 mM) for 24 h on ice. LTA4H was crystallized against reservoir solution containing 10–15% (w/v) PEG 8000, 100 mM NaAc, 100 mM imidazole (pH = 6.0–6.8), and 5 mM YbCl_3_. Crystal growth was accelerated by microseeding as described previously^29^. Crystals were crushed by vortexing in a Seed Bead tube (Hampton Research, Aliso Viejo, CA, USA), and diluted 1:100 into reservoir solution to form the seed stock. The final crystallization drop consisted of 0.7 μL of LTA4H, 0.7 μL of reservoir solution, and 0.2 μL of seed stock over 100 μL of reservoir solution. Crystals were soaked in a reservoir solution containing 30% glycerol before flash freezing.

### Data collection, structure solution and refinement

X-ray diffraction data of LTA4H complexed with bufexamac were collected at 100 K on a MX 225 CCD detector on beamline BL17U at Shanghai Synchrotron Radiation Facility and processed with HKL2000^30^. Data of 180 images were collected with an oscillation range of 1° and a crystal-to-detector distance of 280 mm. The LTA4H structure complexed with bufexamac was solved by molecular replacement with the CCP4 program suite. Coordinates (PDB ID 3FTX) were refined with PHENIX^31^ and Coot^32^. Ramachandran dihedral restraints were employed confirm the quality of the final model. The statistics for data processing and refinement are listed in Table 1.

### Isolation of mouse neutrophils from bone marrow

Female C57bl/c mice aged 6–8 weeks were purchased from Shanghai Laboratory Animal Center, Chinese Academy of Sciences and maintained in a 12 h light/dark cycle. All experimental procedures were implemented according to the guidelines of the IACUC of Shanghai and the National Research Council Guide for the Care and Use of Laboratory Animals. All experimental protocols were approved by the institutional Animal Ethics Committee of East China Normal University. Mouse bone marrow contains a large number of neutrophils^33^. Mouse neutrophils were purified by density gradient centrifugation as described previously^14^,^34^. Mice femurs were obtained from six-to-eight-week-old female C57BL/6 mice, and muscles connected to the bone were removed before immediate soaking in Hanks’ balanced salt solution (HBSS, without calcium and magnesium) supplemented with 100 U/mL penicillin, and 100 U/mL streptomycin. The bones were washed with 75% ethanol once and with sterile PBS twice. Then, the epiphyses were removed. Bone marrow was flushed out by a syringe filled with HBSS containing 2 mM EDTA. After centrifugation (500 × *g* for 10 min), cells were treated with 5 mL 0.2% NaCl followed by 5 mL 1.6% NaCl to remove red blood cells. The remaining cells were resuspended in sterile PBS and layered by Histopaque (Sigma-Aldrich, St. Louis, MO, USA) density gradient. In detail, 3 mL of Histopaque 1119 (density of 1.119 g/mL) was added to a 15 mL conical tube. Then, 3 mL of Histopaque 1077 (density of 1.077 g/mL) overlaid before cell suspension was added. Eventually, cells were centrifuged (834 × *g* for 45 min) and neutrophils at the interface of the two layers were collected with 1 × 10^7 ^cells obtained per mouse.

### Measurement of the release of LTB4 in neutrophil

Neutrophil was suspended in RPMI-1640 medium supplemented with 10% Fetal Bovine Serum (GIBCO, Big Cabin, OK, USA) at 2 × 10^6 ^cells/mL and incubated with bufexamac at the indicated concentration for 30 min at 37 °C, followed by stimulation with 5 μM A23187 for 30 min. The supernatants were soon collected for the measurement of LTB4 release using the EIA kit (Cayman Chemical, Ann Arbor, MI, USA) according to the manufacturer’s instructions.

### Chemotaxis assay

Chemotaxis assay was conducted using a 96-well filter plate with 5 μm pore size polycarbonate membrane (MAMIC 5S10, Millipore, Billerica, MA, USA). Then, 5 μM fMLP (Sigma-Aldrich, St. Louis, MO, USA) was diluted in 27 μL HBSS (without calcium and magnesium) containing 0.5% BSA and placed in the lower chamber. Neutrophils (50 μL; 2 × 10^5^/mL) from mouse bone marrow diluted in the same medium were loaded into the upper wells after being incubated with compounds at the indicated concentrations for 30 min at 37 °C^35^,^36^,^37^. Cells were allowed to migrate towards fMLP at 37 °C for 1 h. The number of migrating cells in the lower chamber was counted using an automated cell counter (Invitrogen, Waltham, MA, USA).

### LPS-induced ALI in mice

Six-to-eight weeks old female C57BL/6 mice were used in this study and randomly divided into the following 5 groups (*n* = 10): (1) control; (2) LPS treated; (3) LPS and dexamethasone (10 mg/kg) treated; (4) LPS and bufexamac (100 mg/kg) treated; and (5) LPS and bufexamac (50 mg/kg) treated. Bufexamac was given orally for seven days before LPS challenge. Afterward, animals were anesthetized and 50 μg LPS from *Escherichia coli* (Sigma-Aldrich, St. Louis, MO,USA) in 40 μL PBS or PBS alone was instilled intratracheally^38^. After 24 h, animals were euthanized to collect lung tissue samples and BALF.

### Bronchoalveolar lavage fluid analysis

Bronchoalveolar lavage was performed by injection of 1 mL PBS followed by gentle infusion^39^,^40^. BALF was centrifuged at 1500 rpm for 10 min. The pellet was resuspended in 100 μL PBS, and total cells were counted using an automated cell counter (Invitrogen, Waltham, MA, USA). The total protein concentration in the supernatant was measured by bicinchoninic acid (BCA) method. Moreover, TNF-α, IL-1β and IL-6 levels in the supernatant were quantified using the ELISA kit (R&D system, USA), and LTB4 level in the supernatant was quantified using a commercial LTB4 EIA kit (Cayman, Minneapolis, MN, USA), according to the manufacturer’s instructions.

### Analysis of lung edema and lung permeability

Dissected lung tissues were weighed immediately and then oven-dried at 60 °C for 48 h. The wet weight and dry weight were both recorded to calculate the wet/dry weight ratio^41^,^42^. Lung permeability was assessed by injecting Evans blue dye (50 mg/kg) into the caudal vein 1 h before euthanasia. The excised lung tissues were photographed immediately to evaluate the extravasated dye into lung tissue^43^.

### Myeloperoxidase assay

MPO activity was assayed to evaluate neutrophil infiltration in LPS-induced ALI mouse lung^2^. Lung tissues were homogenized in potassium phosphate buffer containing 0.5% hexadecyltrimenthyl ammonium bromide. After centrifugation, phosphate buffer (pH = 6.0) containing 0.167 mg/mL *o*-dianisidine hydrochloride and 0.0005% hydrogen peroxide were added to the supernatant. The MPO activity was measured by monitoring the absorbance at 460 nm.

### Transcriptional analysis

Total RNA was isolated by TRIZOL REAGENT (Invitrogen, Waltham, MA, USA) and reverse-transcribed into cDNA using 5×mix RT master (Toyobo, Osaka, Japan), according to the manufacturer’s instruction^44^. qPCR was conducted using the AceQ qPCR SYBR^®^ Green Master Mix (TaKaRa, Japan) according to the manufacturer’s instructions. The primers used in this study are listed in Table 2. The levels of mRNA were determined and normalized by the amount of glyceraldehyde phosphate dehydrogenase (GAPDH).

### Histopathological analysis

Lungs were washed twice with PBS and fixed in 4% paraformaldehyde at 4 °C for 24 h. Then, the lungs were embedded in paraffin and stained with H&E. Pathological changes, such as inflammatory cell infiltration and edema, were observed under Ti–S bright field microscope (Nikon, Melville, NY, USA)^42^.

### Statistical analysis

Data are presented as the means ± standard deviations (SD). Statistical analysis was conducted with GraphPad Prism using Student’s *t* test, one-way analysis of variance (ANOVA), and Bonferroni’s Multiple Comparison Test. A value of *P* < 0.05 was considered significant.

## Additional Information

**Accession codes:** The PDB code: 5BPP (LTA4H and bufexamac complex).

**How to cite this article**: Xiao, Q. *et al.* Bufexamac ameliorates LPS-induced acute lung injury in mice by targeting LTA4H. *Sci. Rep.* **6**, 25298; doi: 10.1038/srep25298 (2016).

## Figures and Tables

**Table 1:** Data collection and refinement statistics of the co-crystallization.

Data collection	
PDB ID	5BPP
Ligand	Bufexamac
Wavelength (Å)	0.97852
Resolution (Å) ^*^	43.59–2.03 (2.08–2.03)
Unit-cell parameters	a = 77.983, b = 87.186, c = 99.434
(Å,°)	α = β = γ =90
Space group	P2_1_2_1_2_1_
Completeness (%)	98.59 (95.90)
R_merge_^a^	6.3 (25.4)
Mean I/δ	37.2 (5.8)
Redundancy	7.4 (7.2)
Refinement	
Resolution (Å)	43.59–2.03
No. of reflections^b^	41873 (2967)
R_work_ (%)^c^	17.20 (21.0)
R_free_ (%)^d^	21.50 (27.20)
R.m.s. deviations	
Bond distance (Å)	0.019
Bond angle (°)	1.893

^a^R_merge_ = Σ_hkl_Σ_i_|l_j_(*hkl*) − 〈l(*hkl*)〉|/Σ_hkl_Σ_i_|l_j_(*hkl*)|, where l_i_(*hkl*) is the intensity of the ith measurement of reflection hkl and 〈l(hkl)〉 is the average intensity this reflection.

^b^Numbers in parentheses are values for the highest-resolution shell.

^c^R_work_ = Σ||F_obs_|-|F_calc_||/Σ|F_obs_|.

^d^R_free_ was calculated with the 5% of reflections set aside randomly throughout the refinement.

**Table 2:** Primer sequences for qPCR.

Gene	Forward sequence	Reverse sequence
TNF-α	CTGTAGCCCACGTCGTAGC	TTGAGATCCATGCCGTTG
IL-1β	ACTCCTTAGTCCTCGGCCA	CCATCAGAGGCAAGGAGGAA
IL-6	TGGAGTCACAGAAGAAGTGGCTAAG	TCTGACCACAGTGAGGAATGTCCAC
GAPDH	TGAAGCAGGCATCTGAGGG	CGAAGGTGGAAGAGTGGGAG
